# Supplementary material for: Dimethylcysteine (DiCys)/o-Phthalaldehyde Derivatization for Chiral Metabolite Analyses: Cross-Comparison of Six Chiral Thiols
Source: Molecules. 2021 Dec 7;26(24):7416. doi: 10.3390/molecules26247416 (PMC8707109; doi:10.3390/molecules26247416)
Supplement: Supplementary file 1 [file molecules-26-07416-s001.zip › molecules-1414331-supplementary.pdf]

Supplementary Information

for

Dimethylcysteine (DiCys)/o-phthalaldehyde derivatization for chiral metabolite analyses: cross-comparison of six chiral thiols

Ankhbayar Lkhagva [1], Hwan-Ching Tai [2*]

[1] Department of Chemistry, National University of Mongolia, Ulaanbaatar, Mongolia

[2] School of Public Health, Xiamen University, Xiamen, Fujian, China

* Corresponding Author

Table S1. Enantiomeric analysis of amino acids using different chiral thiols

(a)

Amino acid	OPA/DiCys	
	Resolution factor	Elution order
Ala	1.7	D, L
Glu	1.2	D, L
Phe	2.3	D, L
Ser	2.8	D, L
Tyr	1.7	D, L

(b)

Amino acid	OPA/NAC	
	Resolution factor	Elution order
Ala	1.1	D, L
Glu	0.6	D, L
Phe	0.3	D, L
Ser	1.6	D, L
Tyr	2.3	L, D

(c)

Amino acid	OPA/NAP	
	Resolution factor	Elution order
Ala	0.9	L, D
Glu	0.5	D, L
Phe	#	
Ser	1.4	D, L
Tyr	2.0	L, D

(d)

Amino acid	OPA/IBLC	
	Resolution factor	Elution order
Ala	2.9	L, D
Glu	1.6	L, D
Phe	2.2	L, D
Ser	2.7	L, D
Tyr	1.8	L, D

(e)

Amino acid	OPA/NBC	
	Resolution factor	Elution order
Ala	#	
Glu	1.6	L, D
Phe	0.7	L, D
Ser	1.4	L, D
Tyr	0.5	L, D

(f)

Amino acid	OPA/ BTCC	
	Resolution factor	Elution order
Ala	#	
Glu	#	
Phe	0.3	L, D
Ser	#	
Tyr	0.9	D, L

Note: Resolution factor (R_S) = 1.18 (t_2- t_1)/(w_2-w_1), where t is elution time, w is the peak width at half maximum height. # indicates failure to resolve the enantiomer pair.

Table S2. MS/MS analysis of chiral amino acids identification in rice water by IBLC/OPA

Amino acid	Retention time (min)	Precursor ion [M+H] (*m/z*)	Manually integrated peak area (MA)	Product ion [M+H-IBLC] (*m/z*)
L-Ala	32.96	379.31	$2.62x10^{7}$	189.07
D-Ala	34.41	379.32	$2.31x10^{6}$	189.07
L-Arg	37.14	464.73	$8.98x10^{6}$	274.13
D-Arg	38.51	464.74	$1.56x10^{6}$	274.15
L-Asn	21.71	422.23	$5.06x10^{6}$	232.09
L-Asp	6.95	423.35	$1.94x10^{7}$	233.10
L-Cys	35.26	411.23	$3.01x10^{5}$	211.03
L-Glu	8.79	437.56	$2.69x10^{7}$	247.08
L-Gln	27.62	436.34	$2.31x10^{6}$	246.10
Gly	31.12	365.01	$9.67x10^{6}$	175.06
L-His	29.47	445.09	$1.95x10^{6}$	255.16
L-Ile	43.36	421.14	$9.29x10^{6}$	231.08
L-Leu	44.87	421.43	$4.32x10^{6}$	231.08
L-Lys	42.05	436.41	$1.08x10^{6}$	246.10
D-Lys	43.93	436.40	$3.31x10^{5}$	246.10
L-Met	41.24	439.04	$2.99x10^{6}$	249.05
L-Phe	41.99	455.39	$4.91x10^{6}$	265.08
D-Phe	43.12	455.38	$4.66x10^{5}$	265.08
L-Pro	46.70	405.08	$4.26x10^{6}$	215.09
L-Ser	23.52	395.31	$4.55x10^{6}$	205.07
D-Ser	24.92	395.33	$4.75x10^{5}$	205.07
L-Thr	30.17	409.13	$1.44x10^{7}$	219.08
L-Trp	42.04	494.28	$2.95x10^{6}$	304.05
L-Tyr	36.39	471.45	$3.42x10^{6}$	281.10
D-Tyr	37.63	471.41	$4.20x10^{5}$	281.10
L-Val	40.85	407.04	$7.68x10^{6}$	217.09
D-Val	41.61	407.03	$2.46x10^{5}$	217.08

Table S3. MS/MS analysis of chiral amino acids identification in rice water by DiCys/OPA

Amino acid	Retention time (min)	Precursor ion [M+H] (*m/z*)	Manually integrated peak area (MA)	Product ion [M+H-DiCys] (*m/z*)
L-Ala	32.51	337.41	$1.75x10^7$	189.07
D-Ala	29.50	337.42	$1.54x10^6$	189.07
L-Arg	34.27	422.02	$5.99x10^6$	274.13
D-Arg	33.67	422.03	$1.04x10^6$	274.13
L-Asn	16.05	380.21	$3.37x10^6$	232.09
L-Asp	6.54	381.12	$1.29x10^7$	233.10
L-Cys	33.09	369.23	$9.50x10^5$	221.28
L-Glu	7.68	395.45	$1.79x10^7$	247.08
L-Gln	55.81	394.32	$1.54x10^6$	246.10
Gly	22.51	323.08	$6.44x10^6$	175.06
L-His	24.88	403.43	$1.30x10^6$	255.16
L-Ile	40.88	379.21	$6.19x10^6$	231.08
L-Leu	41.07	379.26	$2.88x10^6$	231.08
L-Lys	45.81	394.09	$7.21x10^5$	246.10
D-Lys	44.17	394.08	$2.21x10^5$	246.10
L-Met	35.68	397.32	$1.99x10^6$	249.05
L-Phe	43.22	413.12	$3.27x10^6$	265.08
D-Phe	42.14	413.13	$3.11x10^5$	265.08
L-Pro	24.39	363.33	$2.84x10^6$	215.09
L-Ser	17.43	353.06	$3.03x10^6$	205.07
D-Ser	16.31	353.07	$3.17x10^5$	205.07
L-Thr	29.22	367.13	$9.60x10^6$	219.08
L-Trp	38.82	452.24	$1.96x10^6$	304.05
L-Tyr	37.13	429.21	$2.28x10^6$	281.10
D-Tyr	35.93	429.23	$2.80x10^5$	281.10
L-Val	36.52	365.43	$5.12x10^6$	217.09
D-Val	35.29	365.42	$1.64x10^5$	217.08

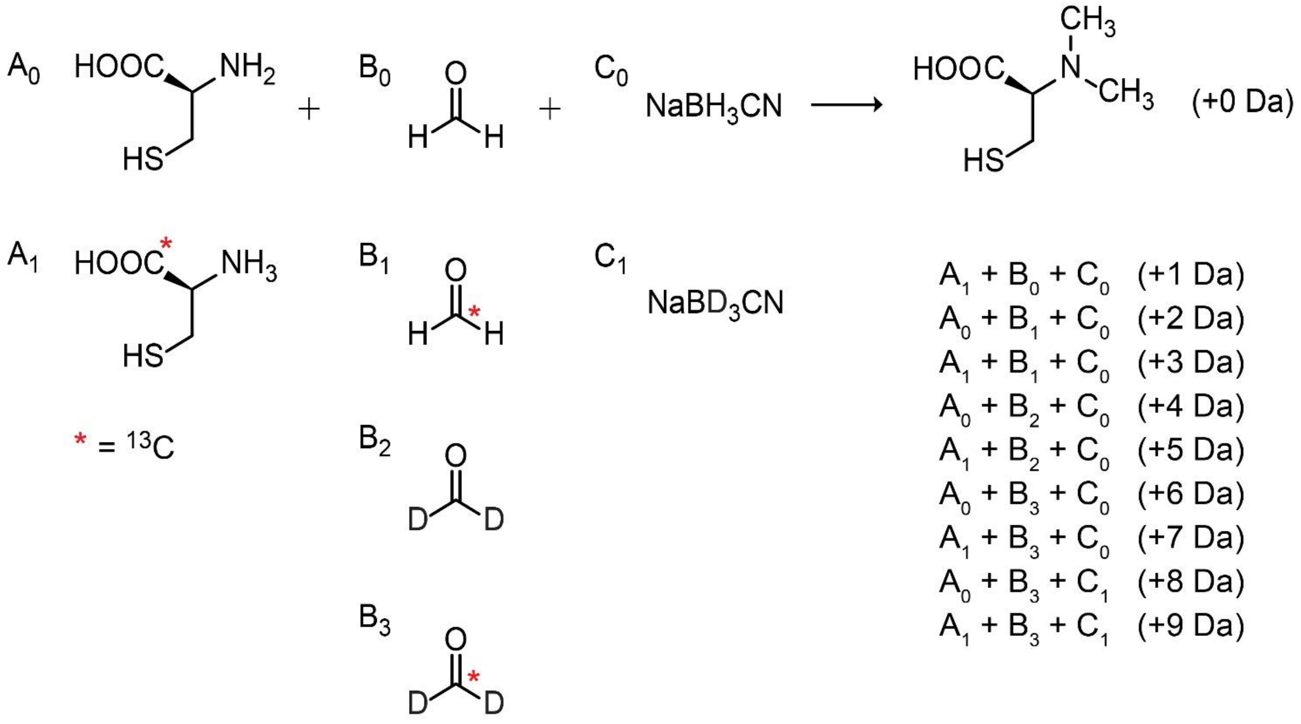

Figure S1. Proposed synthesis scheme for isotope-tagged DiCys.

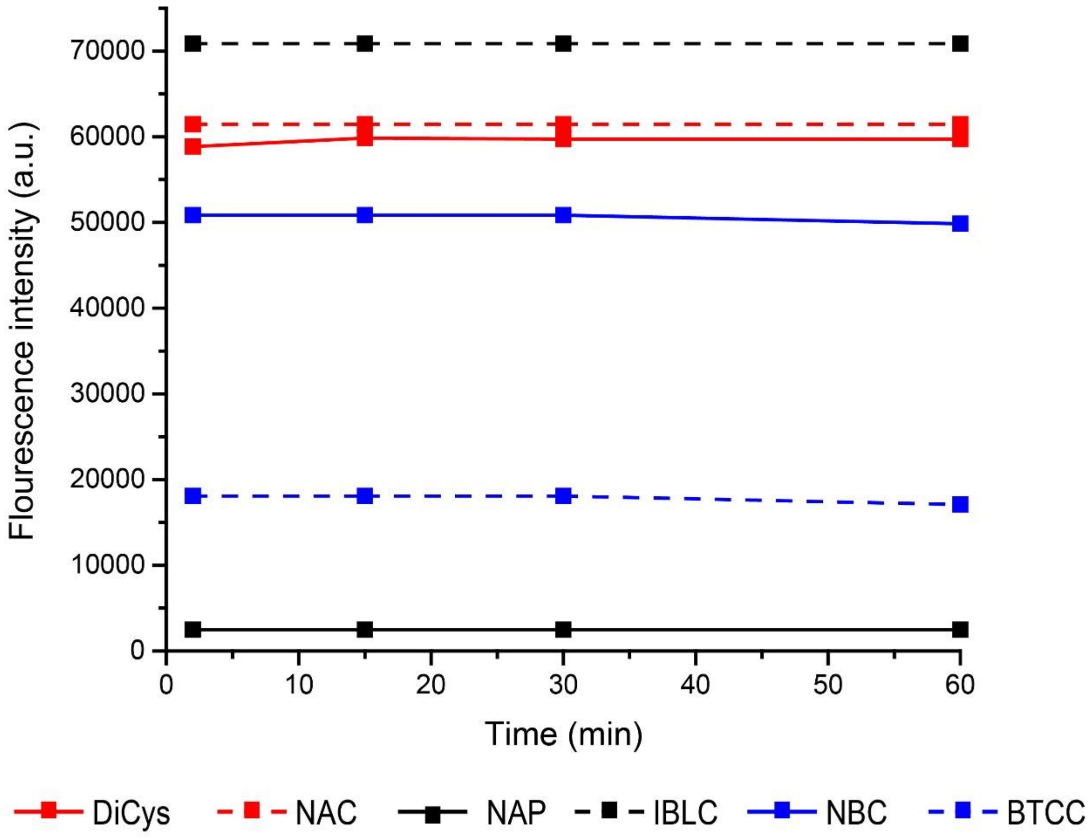

Figure S2. The stability of OPA-derivatized alanine with different thiols at 4 ºC.

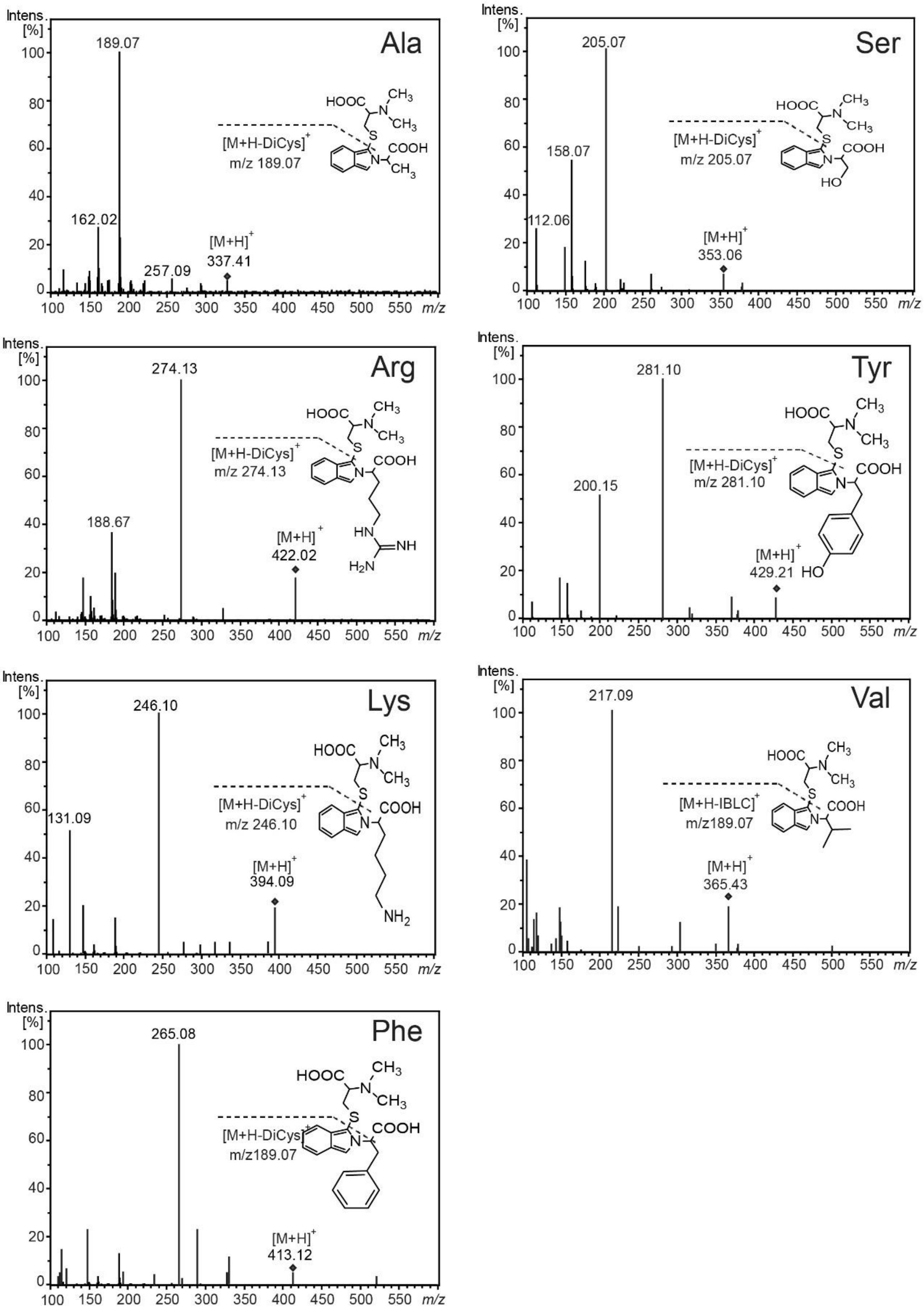

Figure S3. Fragmentation patterns of DiCys/OPA adducts with seven D-amino acids

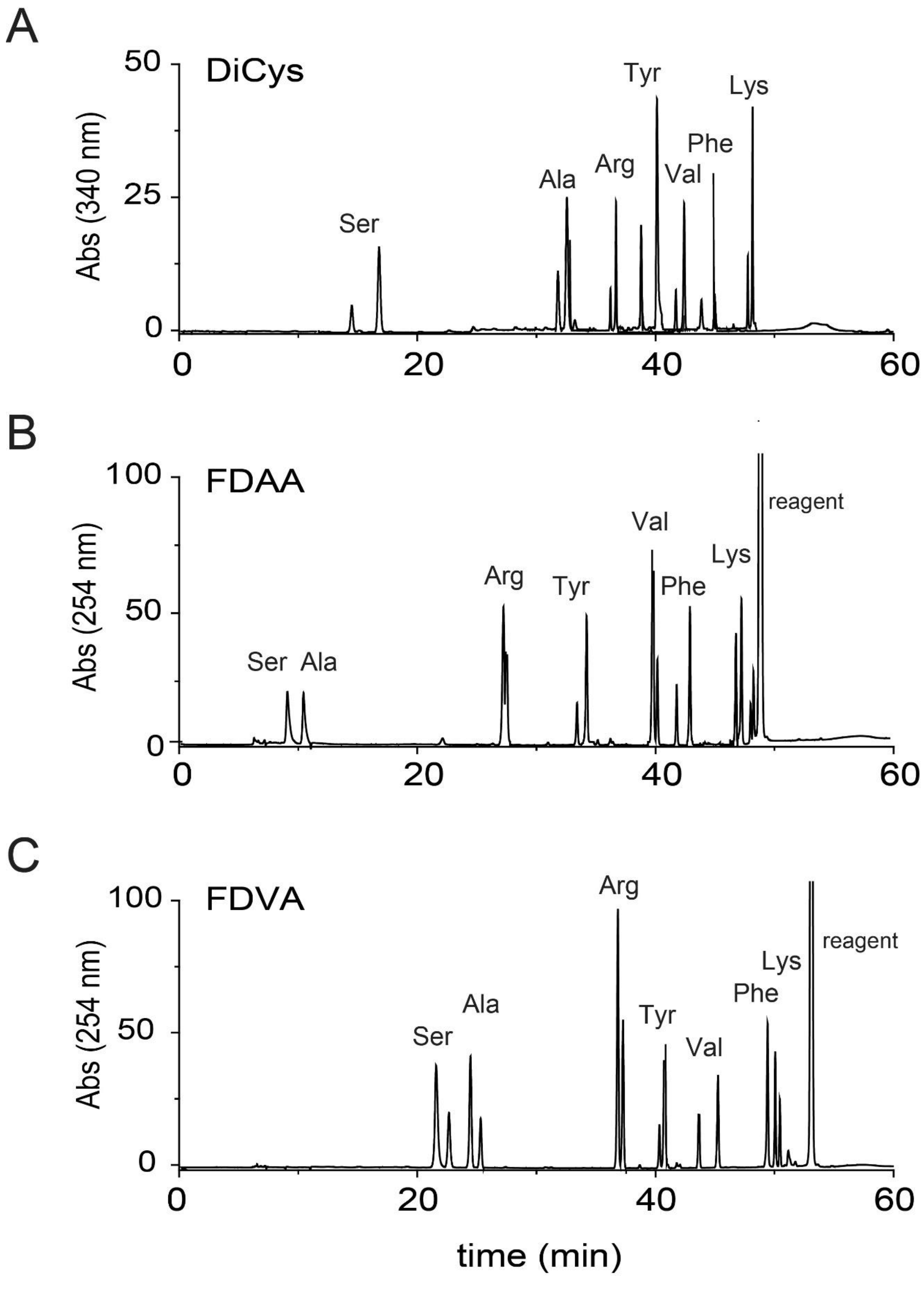

Figure S4. RP-HPLC analysis of amino acid enantiomers derivatized with OPA/DiCys (A) and Marfey's reagents—FDAA (B), and FDVA (C). The ratio between L: D amino acids is 3:1.
